# Time to Act for Clean Air for All in the WHO Eastern Mediterranean Region; Strategic Actions for the Health Sector

**DOI:** 10.3389/ijph.2024.1608001

**Published:** 2024-10-22

**Authors:** Heba Safi, Mazen Malkawi, Aurelio Tobías, Massimo Stafoggia, Sophie Gumy

**Affiliations:** ^1^ Climate Change, Health and Environment Unit, Healthier Population Department, WHO Regional Office for the Eastern Mediterranean, Amman, Jordan; ^2^ Institute of Environmental Assessment and Water Research (IDAEA), Spanish Council for Scientific Research (CSIC), Barcelona, Spain; ^3^ Department of Epidemiology, Lazio Region Health Service/Azienda Sanitaria Locale (ASL) Roma 1, Rome, Italy; ^4^ Department of Environment, Climate Change and Health, World Health Organization, Geneva, Switzerland

**Keywords:** air pollution, health, WHO air quality guidelines (AQG), particulate matter, air pollutants

## Introduction

The WHO Eastern Mediterranean Region (EMR), encompassing nearly 745 million people across 22 countries, faces significant environmental health challenges [1, 2]. Every year, environmental health risks cause more than one million premature deaths across the Region. Air pollution alone accounts for more than 560,000 premature deaths, with 370,000 attributed to ambient air pollution [3]. These deaths are due to five main health outcomes, including ischemic heart disease (IHD), stroke, chronic obstructive pulmonary diseases (COPD), lung cancer (LC), and acute lower respiratory infections (ALRI) [4]. There is a strong belief that these figures are underestimated due to insufficient epidemiological studies on other health outcomes, for which the causality and evidence are still evolving.

In 2019, the region recorded the second highest annual population-weighted exposure of particulate matter with aerodynamic equal to or less than 2.5 μm (PM_2.5_), with an average concentration of 43.3 μg/m^3^ - nine times higher than WHO Air Quality Guideline (AQG) values [3]. In addition, the region had the highest annual concentrations of nitrogen dioxide (NO_2_), with an average of 47.5 μg/m^3^, among WHO regions, surpassing WHO AQG values by nearly five times [5]. The region’s air quality is extremely affected by natural sources, i.e., sand and dust storms (SDS), which contribute 30%–60% of the total PM levels across various EM countries [6, 7]. However, anthropogenic sources such as unsustainable development, continued urbanization, industrialization, transportation, open burning of municipal and agricultural waste, and specific sources, i.e., diesel generators, are considerable and should be addressed.

The WHO AQG 2021 indicates that air pollution has detrimental health impacts at all exposure levels, even at the lowest concentrations. A critical message of the guidelines is that each reduction in outdoor concentrations of key air pollutants yields health benefits for the exposed population [4]. This is a wake-up call to reconsider current air quality management strategies for health protection.

## Air Quality and Health in the Region

**FIGURE 1:**
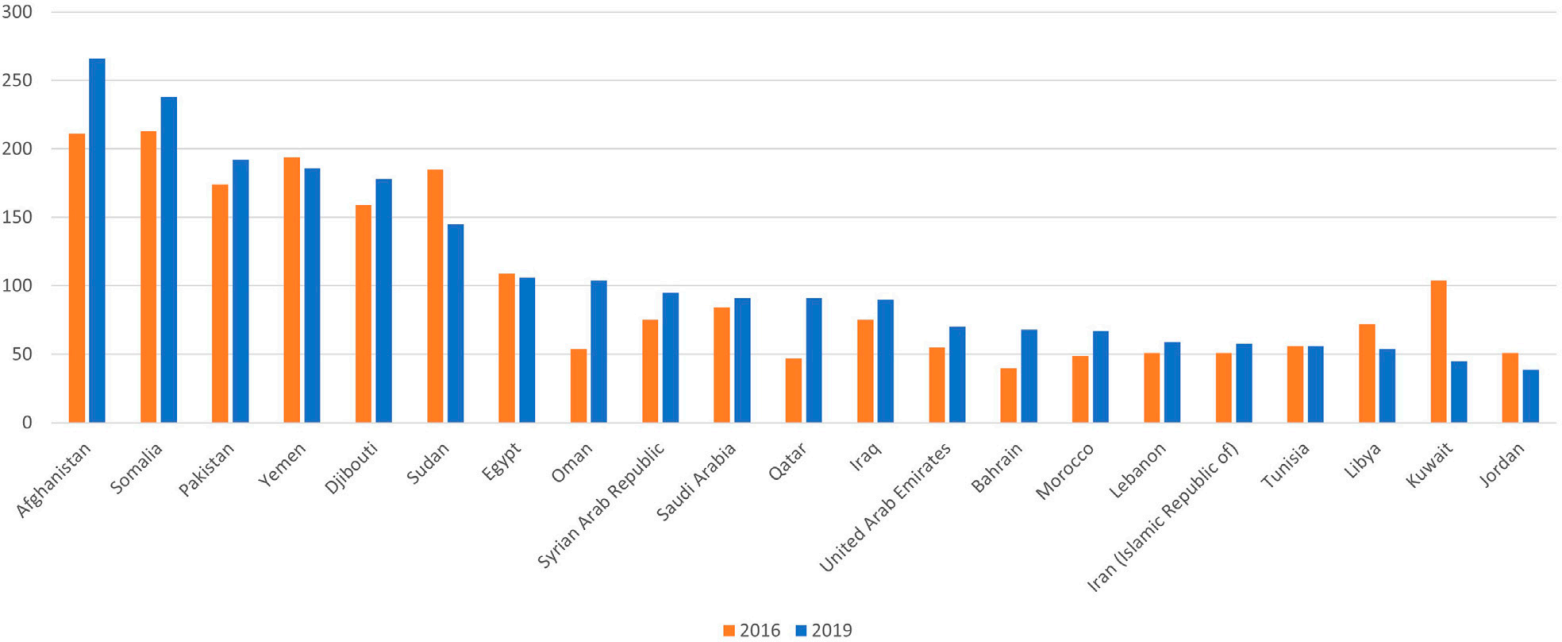
Age-standardized death rate attributable to air pollution for each Eastern Mediterranean (EM) country in 2016 and 2019 (Eastern Mediterranean Region, 2024).

**FIGURE 2:**
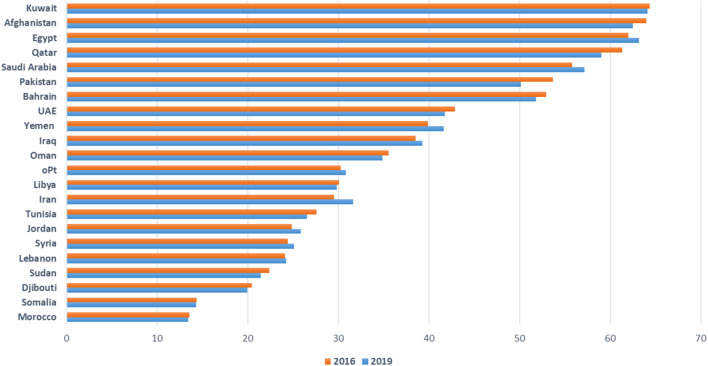
Population-weighted exposure to Particulate Matter (PM2.5) in 2016 and 2019 by Eastern Mediterranean (EM) country (Eastern Mediterranean Region, 2024).

The region is off-track; the air pollution-attributable death rate increased from 72.8 per 100,000 inhabitants in 2016 to 77.6 per 100,000 in 2019 [3]. In 15 countries in the region, the age-standardized mortality rate worsened in 2019 compared to 2016 (Figure 1). In only five countries the age-standardized mortality rate had slightly improved. Although 17 out of 22 countries in the region have national ambient air quality standards (NAAQS) for the key air pollutants, PM_2.5_ levels were steady with no significant reduction between 2016 and 2019 (Figure 2) [3, 8]. This stagnation indicates inadequate non-health-based air quality management strategies, plans, and regulatory enforcement.

## Air Quality Management in the Region

A regional survey to assess the countries’ capacities in air quality monitoring and management systems in the Region was conducted through the WHO/EMR/HPD/CHE. Responses to survey were received from 16 out of 22 EM countries [Afghanistan, Egypt, Islamic Republic of Iran, Iraq, Jordan, Kuwait, Lebanon, Morocco, Pakistan, Occupied Palestinian Territory (oPt), Saudi Arabia, Somalia, Sudan, Syria, Tunisia and Yemen]. According to the survey, 11 countries recognize the right to clean air, and have national or subnational air quality action plans (AQAPs). In eight countries (Egypt, Iran, Jordan, Kuwait, Lebanon, Morocco, Saudi Arabia, and Tunisia) the health component is included into the national AQAPs, and its implementation is a shared responsibility between ministries of environment and health. In Afghanistan, Iraq, and Pakistan only environmental authorities are responsible for AQAPs implementation. In Somalia, Syria, and Yemen where are no AQAPs, there are some fragmented efforts to tackle air pollution. Even though air quality management is a multi-stakeholder multi-sectoral public health issue, health sector involvement in air quality management remains limited across the Region.

## Strategic Actions for Health Sector

The health sector through leadership and intersectoral governance, evidence-based advocacy, operational programmes, surveillance, and monitoring can drive progress in tackling air pollution and obtaining short- and long-term health benefits. This includes:

### Advocate for Action by Other Sectors to Reduce Air Pollution [Adopt Health in All Policies (HiAP) Approach]

While the health sector works to minimize its own emissions of key air pollutants, it is crucial to advocate for actions aimed at improving air quality beyond the health sector, such as energy, transport, housing, labor, industry, food systems and agriculture, power generation, waste management, water and sanitation, and urban planning. The health sector plays an important role in integrating health considerations into air quality policies by assessing the associated health and economic impacts of action and inaction. In addition to defining and promoting national indicators to measure progress in air quality management policies, interventions, and strategies.

### Address the Root Causes of Disease

Reducing the annual number of 560,000 premature deaths associated with air pollution requires the efficient scale-up of a primary prevention strategy. Integrating air quality measures into disease prevention programmes, especially those targeting non-communicable diseases (NCDs) is essential. According to the global strategy to prevent non-communicable diseases, healthy environments, such as clean air, healthy and safe work environments, and chemical safety, are key elements in NCDs prevention, and relevant action is being called for.

### Building the Capacities of the Health Sector

The health workforce needs regular training to better understand the health risks of exposure to indoor and outdoor air pollution, communicate health risks, and advise patients and vulnerable populations on personal measures to mitigate health risks from air pollution. In addition, health workforce needs skills to leverage the “health argument” for scaling up actions, engage in high-level discussions and intersectoral dialogues, monitor economic and environmental investments, and communicate health impacts with all concerned stakeholders and community.

### Enhancing Surveillance and Early Warning and Alerting Systems

A robust health surveillance system is crucial for conducting health impact assessment, advancing air pollution and health research, and developing an impact-based people-centered early warning and alert system. A well-established early warning and alert system ensures that timely and actionable health messages are delivered directly to the public. Through this, the health sector empowers individuals to take proactive measures to reduce public exposure to air pollution and its health impacts while shaping policy decisions.

### Conclusion

Reducing air pollution levels in the WHO EMR hinges on a robust response from the health sector. This includes advocating for actions beyond the health sectors through integrating and prioritizing associated health implications, scaling up primary prevention strategies, empowering the health workforce, and strengthening health surveillance and early warning and alert systems. By driving these strategic actions, the health sector can play a crucial role in mitigating air pollution’s impact and advancing public health across the Region.
